# Amniotic Membrane Transplantation for Persistent Epithelial Defects and Ulceration due to *Pseudomonas* Keratitis in a Rabbit Model

**DOI:** 10.18502/jovr.v16i4.9744

**Published:** 2021-10-25

**Authors:** Mohammad Mehdi Soltan Dallal, Farhad Nikkhahi, Seyed Mostafa Imeni, Saber Molaei, Seyed Kazem Hosseini, Zohreh Kalafi, Sara Sharifi Yazdi, Hedroosha Molla Agha Mirzaei

**Affiliations:** ^1^Division of Microbiology, Department of Pathobiology, School of Public Health, Tehran University of Medical Sciences (TUMS), Tehran, Iran; ^2^Food Microbiology Research Center, Tehran University of Medical Sciences, Tehran, Iran; ^3^Medical Microbiology Research Center, Qazvin University of Medical Science, Qazvin, Iran; ^4^Biodiversitat, Ecología, Technologia Ambiental i Alimentaria )BETA Tech Center(, (TECNIO Network), U Science Tech, University of Vic-Central University of Catalonia, Carrer de la Laura 13, 08500 Vic, Spain; ^5^AJA University of Medical Sciences, Tehran, Iran; ^6^Quality Control Manager of Iranian Tissue Bank Research & Preparation Center, Director of Stem Cells Preparation Unit, Tehran University of Medical Sciences, Tehran, Iran; ^7^School of Medicine, Tehran University of Medical Sciences (TUMS), Tehran, Iran; ^8^Food Microbiology Research Center, Tehran University of Medical Sciences, Tehran, Iran

**Keywords:** Ciprofloxacin, Human Amniotic Membrane, Keratitis, Pseudomonas aeruginosa, Rabbit

## Abstract

**Purpose:**

The use of amniotic membrane has been suggested in the treatment of infectious keratitis for its intrinsic anti-infective properties probably mediated by its anti-inflammatory effects. The aim of this study was to investigate the effect of amniotic membrane transplantation (AMT) along with ciprofloxacin to cure the primary stages of *Pseudomonas *keratitis.

**Methods:**

In total, 28 rabbits were selected and divided in four groups as follows: group 1 as control, group 2 with amniotic membrane, group 3 with ciprofloxacin, and group 4 with amniotic membrane combined with ciprofloxacin. About 0.05 cc suspension of *Pseudomonas* *aeruginosa*, 27853 ATCC was injected into corneal stroma.

**Results:**

The results showed groups of AMT, AMT + ciprofloxacin, and ciprofloxacin had 0% perforation while the control group had 85.6%. Average infiltration of 5.5 mm was observed in ciprofloxacin group, 5 mm in AMT + ciprofloxacin group, 24 mm in AMT group, and finally 23.75 mm for control. Amniotic membrane showed to be effective in prevention of cornea perforation as well as remission of *Pseudomonas *keratitis. There was no significant difference between ciprofloxacin groups in comparison with ciprofloxacin + AMT group. However, regarding the anti-inflammatory effect, the process of improvement of inflammation in ciprofloxacin + AMT group was faster.

**Conclusion:**

Transplantation of amniotic membrane in the primary stages of *Pseudomonas* keratitis treatment remarkably prevents the disease and it can be used to control its process.

##  INTRODUCTION 

Human amniotic membrane (AM) forms the inner wall of the membranous sac that surrounds and protects the embryo during gestation. It consists of a single layer of ectodermally derived columnar epithelial cells attached to a basement membrane with an underlying layer of mesenchyme.^[1]^ Amniotic membrane transplantation (AMT) is widely used in various ocular surface diseases such as neurotrophic keratitis and persistent epithelial defects,^[2,3]^ band keratopathy,^[4]^ bullous keratopathy,^[5,6]^ after excimer laser photorefractive keratectomy,^[7,8]^ after the excision of a conjunctival mass,^[9,10]^ pterygium,^[11,12]^ ocular surface reconstruction in symblepharon,^[13,14]^ acute chemical injury,^[15,16]^ and a chronic limbal deficiency.^[17,18]^ When used as a graft (epithelial side up), AM is expected to become incorporated in the recipient tissue. If it is used as a patch (epithelial side down), it works as a biological bandage affording a cover for a limited duration or a combination of these. The use of AM has been also suggested in the treatment of infectious keratitis because of its intrinsic anti-infective properties probably mediated by its anti-inflammatory effects and because AM may act as a long-term drug delivery system.^[19,20,21]^ The antimicrobial effects of AM have been demonstrated against several species such as *Escherichia coli*, Group A *Streptococci*, *Pseudomonas aeruginosa,* and *Staphylococcus aureus*.^[22,23]^ AM graft for epithelial reformation has been employed in order to eradicate the *P. aeruginosa* infection of keratitis and have exhibited desirable outcomes.^[24]^

The aim of the current study was to investigate the effect of AMT along with ciprofloxacin to cure the primary stages of *Pseudomonas *keratitis.

##  METHODS 

All experiments were carried out in accordance with the UK Animals (Scientific Procedures) Act, 1986 and associated guidelines, EU Directive 2010/63/EU for animal experiments. In addition, this study is certified by accreditation research ethics national committee with issue code of IR.TUMS.REC.1394.2091.

**Figure 1:**
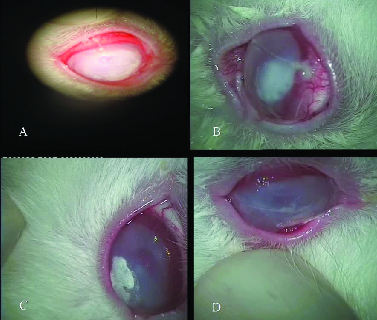
Cornea infiltration of the four groups of experiment. (A: Control group; B: AMT group; C: ciprofloxacin; D: AMT + ciprofloxacin).

In total, 28 male rabbits with an average weight of 1.5–2 kg were selected. The AM was prepared according to Song and Kim method.^[25]^ Human placenta was obtained after an elective caesarean section in a woman who was seronegative for human immunodeficiency virus, hepatitis B, C, and syphilis. Under a lamellar flow hood, the placenta was first washed free of blood clots with sterile saline. The inner AM was separated from the rest of the chorine by blunt dissection and flattened onto a nitrocellulose membrane.^[26]^ The membrane with the filter was then washed three times with phosphate buffered saline (PBS) containing 50 μg/ml penicillin, 50 μg/ml streptomycin, and 2.5 μg/ml amphotericin B and put in M199 culture for 24 hr with antibiotic solution including streptomycin, cloxacillin, ceftriaxone, and amphotericin B, and finally packed in pieces of 1.5 × 1.5 in three of sterilized nylon and stored in –80ºC in freezer. Twenty-eight rabbits were divided into four groups as follows: group 1 as control group [Figure 1A], group 2 as AM [Figure 1-B], group 3 as ciprofloxacin [Figure 1C], and group 4 as AM combined with ciprofloxacin [Figure 1D].

The rabbits were anesthetized with intramuscular injection of ketamine hydrochloride (30 mg/kg) and xylazine hydrochloride (5 mg/kg) and then a drop of tetracaine HCL 0.5% was applied to the rabbits' right eye. About 0.05 cc suspension of *P. aeruginosa* 27853 ATCC was injected into corneal stroma with a sterile 30 G needle connected to a micro-syringe, using an operating microscope. The experimental keratitis was allowed to proceed untreated for 20 hr. There was no interference in the control group. In groups 3 and 4, the AM in pieces of 1.5 × 1.5 cm transplanted to the entire corneal surface by eight interrupted 10.0 nylon sutures. On the first day, ciprofloxacin drop was injected to groups 2 and 4 every 30 min.

On the second day to seventh day every 2 hr, the results were registered in aspect of perforation in cornea and the amount of infiltration by the use of image J 1 software.

##  RESULTS 

Results were registered as clinical reports on the first, third, and seventh day [Table 1]. During the first 20 hr, after injecting *P. aeruginosa,* a white opacity appeared in all rabbits. Rabbits had corneal ulcers and on the second day, the conjunctiva was markedly hyperemic in four groups. At the end of the first week, Hypopyon formation was noticed in five eyes in the AMT group. Corneal perforation was noticed in four cases in the control group but in no case in other groups.

**Table 1:** Clinical results of four groups on the first, third, and seventh day and in the first 20 hr*


**Groups/days**	**First day**	**Third day**	**Seventh day**
**Control**	Average infiltration 2.25 mm with corneal opacity and without epithelial defect	Average infiltration 7.01 mm with corneal opacity, epithelial defect, and descemeto cell	Average infiltration 23.75 mm with corneal opacity and epithelial defect
**AMT**	Average infiltration 2.62 mm and without epithelial defect	Average infiltration 6.25 mm and without epithelial defect	Average infiltration 24 mm and without epithelial defect, melting, and scar
**ciprofloxacin**	Average infiltration 2.35 mm and without epithelial defect	Average infiltration 4.51 mm and epithelial defect	Average infiltration 5.5 mm, epithelial defect, and scar with diameter 2.5 × 2.5 mm
**ciprofloxacin + AMT**	Average infiltration 2.6 mm and without epithelial defect	Average infiltration 4 mm and without epithelial defect	Average infiltration 5 mm and without epithelial defect and scar
*Findings in the first 20 hours only the first day for all groups **AMT, amniotic membrane transplantation			

**Table 2:** Clinical outcomes of perforation and infiltration in all four groups of *Pseudomonas* keratitis


**Results**	AMT*	**AMT + ciprofloxacin**	**ciprofloxacin**	**Control**
Perforation	(0%) 0	(0%) 0	(0%) 0	(85.7%) 6
Infiltration	24 mm	5 mm	5.5 mm	23.75 mm
Significant difference compared to control group (*P*-value)	*P* < 0.5	*P* < 0.5	*P* < 0.5	–
*AMT, amniotic membrane transplantation				

The results showed that the cornea had infiltration in which central part, an area with the size of 6 mm had descemeto cell and progress toward causing perforation in cornea control group (A: Control group). On the other hand, AMT group conjunctiva inflammation showed to be less than control group and in cornea examination the amount of infiltration showed to be 24 mm (B: AMT group). For ciprofloxacin group, ciprofloxacin sediment was clearly visible (C: ciprofloxacin) and finally in the group with ciprofloxacin + AMT as well as the ciprofloxacin group the decrease of opacity cornea was quite visible (D: AMT + ciprofloxacin).

The clinical results of four groups in examination of *Pseudomonas *keratitis are also shown in Table 2. These results showed that AMT + ciprofloxacin group had 0% perforation and the control group had 85.7%. Average infiltrations were 5 mm in AMT + ciprofloxacin groups and 23.75 mm in control.

The result showed that the AMT, ciprofloxacin, and AMT + ciprofloxacin were effective on perforation and infiltration on all groups compared to the control group.

In addition, there was no significant difference between ciprofloxacin group and ciprofloxacin combination with AM. In the same way, there was no significant difference between membrane amniotic group and ciprofloxacin compared with combined group of ciprofloxacin with membrane amniotic.

##  DISCUSSION 

Few studies have investigated the effect of AMT in the surgical treatment of severe infectious keratitis with corneal ulceration or perforation.^[19,23,27]^ The main advantages of AMT in the treatment of bacterial keratitis that we observed are the epithelial bandage properties, which allowed early use of topical steroids; the anti-inflammatory and anti-scarring effects of the AM, the promotion of epithelialization, and the possible benefits of a direct antimicrobial role of the AM.^[28]^ These studies found AMT to be effective in treating neurotrophic ulcer, inflammatory corneal ulcer, bullous keratopathy, inflammatory or non-inflammatory scleral ulcer and as an adjuvant treatment of pterygium excision. The basement membrane of an AM promotes epithelial growth and differentiation, reinforces the adhesion of basal epithelial cells and prevents epithelial apoptosis. The stroma matrix suppresses TGF-b signaling, proliferation, and myofibroblastic differentiation of normal human corneal and limbal fibroblasts and thus, inhibiting the unwanted production of extracellular matrix and scarring.^[6]^ These properties have made the AM an ideal reconstructive substrate for repairing persistent epithelial defects and corneal ulcers,^[2]^ conjunctival defect,^[9,10]^ chemical or thermal injury,^[15,29]^ and limbal cell deficiency.^[16,17]^ The application of AM in the treatment of corneal perforation and scleromalacia has been also reported.^[30,31]^ The human AM possesses anti-inflammatory, antifibrotic, and antiangiogenic properties, and these attributes make it ideal for ocular surface reconstruction procedures.^[32,33]^ In addition, the AM also has antimicrobial properties due partly to its anti-inflammatory effects, and also due to secretion of elfin and secretory leucocyte proteinase inhibitor, both of which have antimicrobial actions and act as components of the innate immune system.^[34,35]^ It also contains cystatin E, an analogue of cysteine proteinase inhibitors, which has complementary antiviral properties.^[36]^ Furthermore, AM transplantation (AMT) is reserved for cases of postinfectious ulcers after an appropriate period of anti-infective treatment when clinical signs are improving.^[37]^ This is because the anti-infective properties of AM are nonspecific and not considered to be potent enough to be effective in acute infective keratitis. This is the reason behind the concept of fortifying AM with antimicrobial drugs to make it a viable therapeutic modality in the setting of active infections of the cornea. Antibiotic-impregnated medical devices such as catheters, bone and cardiac implants have been in use for over a decade.^[22,38]^ Various studies have shown the potential of such an approach, for example, in vascular surgery and arthroplasty, where they appear effective in reducing the risk of bloodstream infections or in limiting deep wound infections.^[39,40]^ AM is effective in remission of *Pseudomonas *keratitis and prevention of cornea perforation and controlling as well as anti-*Pseudomonas *effects. There was no difference between ciprofloxacin group in comparison with ciprofloxacin + AMT group. However, regarding anti-inflammatory effects, the process of improvement of inflammation in ciprofloxacin + AMT group was faster. During this research, we came to conclusion that transplantation of AM in the primary stages of *Pseudomonas* keratitis treatment remarkably prevents the disease and it can be used to control its process.

##  Financial Support and Sponsorship

Nil.

##  Conflicts of Interest

There are no conflicts of interest.
